# Food resource uncertainty shapes the fitness consequences of early spring onset in capital and income breeding migratory birds

**DOI:** 10.1002/ece3.9637

**Published:** 2022-12-21

**Authors:** Anna Ejsmond, Maciej Jan Ejsmond

**Affiliations:** ^1^ Department of Biological Sciences University of Bergen Bergen Norway; ^2^ Research Centre Snæfellsnes University of Iceland Stykkishólmur Iceland; ^3^ Department of Arctic Biology University Centre in Svalbard Longyearbyen Norway; ^4^ Institute of Environmental Sciences Jagiellonian University Cracow Poland

**Keywords:** capital breeding, eider, income breeding, migratory birds, nesting onset, recruitment, stochastic fluctuations, temporally fluctuating resource gain

## Abstract

Due to climate change, the timing of spring arrival and nesting onset in many migratory bird species have advanced. Earlier spring onsets prolong the available breeding period but can also deteriorate local conditions, leading to increased temporal variation in resource availability. This interaction between phenological shifts in nesting onset and short‐term temporal variation in food gain has unknown consequences for fitness of migratory bird species. We model two contrasting breeding strategies to investigate the fitness consequences of stochastically fluctuating food gain and storing of energetic reserves for reproduction. The model was inspired by the biology of common eiders (*Somateria mollissima*), which store extensive reserves prior to egg laying and incubation (capital breeding strategy), and king eiders (*S. spectabilis*), which continue to forage during nesting (income breeding strategy). For capital breeders, foraging prior to breeding increases energy reserves and clutch size, but for both strategies, postponing nesting reduces the chances of recruitment. We found that in scenarios with early spring onset, the average number of recruits produced by capital breeders was higher under conditions of stochastic rather than deterministic food gain. This is because under highly variable daily food gain, individuals successful in obtaining food can produce large clutches early in the season. However, income breeders do not build up reserve buffers; consequently, their fitness is always reduced, when food availability fluctuates. For both modeled strategies, resource uncertainty had only a minor effect on the timing of nesting onset. Our work shows that the fitness consequences of global changes in breeding season onset depend on the level of uncertainty in food intake and the degree to which reserves are used to fuel the reproductive effort. We predict that among migratory bird species producing one clutch per year, capital breeders are more resilient to climate‐induced changes in spring phenology than income breeders.

## Graphical abstract

The common eider females with their ducklings leaving nesting site in a high Arctic colony. These capital breeders do not feed during the time of egg incubation. Hence, females need to gain extensive body reserves prior to breeding. Photo credit: Anna Ejsmond.

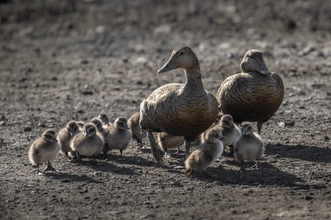

## 1 INTRODUCTION

Climate change has forced migratory species to alter the timing of seasonal behaviors (Parmesan, 2006; Thackeray et al., 2016; Zurell et al., 2018). In response to earlier spring onset, many birds have begun arriving at breeding grounds or initiating their nesting earlier (Carey, 2009; Conklin et al., 2021; Zurell et al., 2018). This prolonged breeding season relaxes the time constraints on the development of fledglings, which can benefit successful offspring migration and recruitment (Drent et al., 2003; Morrison et al., 2019; Verhulst & Nilsson, 2008). However, earlier spring onset is often correlated with increased spatiotemporal variation in resource availability and predation rates that offset the fitness benefits of the prolonged breeding season (Kwon et al., 2019; Layton‐Matthews et al., 2020; Saalfeld et al., 2019; Visser & Gienapp, 2019). Thus, despite the earlier arrival to nesting sites, many migratory bird species have shown reduced breeding success in response to recent climatic changes (Bay et al., 2018; Grimm et al., 2015; Layton‐Matthews et al., 2020; Zurell et al., 2018). This is because timing of migration is often influenced by large‐scale climatic cues, whereas local conditions determine breeding success (Carey, 2009). Hence, short‐term temporal variation in resource availability at breeding grounds should be considered one of the most important determinants of the fitness response to climate‐induced shifts in the spring phenology of migratory birds.

Capital breeders use the time before nesting to amass energetic reserves that subsequently contribute to reproduction, whereas income breeders depend on concurrent resource acquisition (Drent & Daan, 1980; Jönsson, 1997). In life history models of birds that produce a single clutch per year, these breeding strategies produce contrasting changes in clutch size in response to the prolonged breeding season (Ejsmond et al., 2021). Specifically, capital breeders are expected to produce smaller clutches with increased offspring quality, whereas for income breeders, the longer the breeding season the greater the clutch size and offspring quality (Ejsmond et al., 2021). The breeding strategies of birds are distributed along a continuum from pure capital breeding and income breeding strategies, with many species of ducks, geese, swans, waders, gulls, penguins, flamingos, owls, and some passerines relying on internal reserves as a significant proportion of resources used for reproduction (Drent et al., 2006; Hobson & Jehl, 2010; Krapu, 1981; Kullberg et al., 2005; Langin et al., 2006; Mawhinney et al., 1999; Nolet, 2006; Poisbleau et al., 2015; Rendon et al., 2011; Solonen, 2014; Yates et al., 2010).

Internal reserves provide a buffer that helps birds cope with temporal stochastic fluctuations in food gain. According to life history theory, the degree of capital breeding usually increases with stochastic fluctuations in food availability prior to and during the breeding season (Fischer et al., 2011; Stephens et al., 2014). However, little is known about changes in fitness of capital and income breeders in conditions of fluctuating food availability, temporally constrained breeding and changing breeding season durations. Consequently, it is difficult to predict the responses of migratory birds to the interaction between stochastic food intake and the prolonged breeding season observed in many ecosystems around the world (Carey, 2009; Zurell et al., 2018).

Due to predicted changes in snow and sea ice‐melt dates, high‐latitude Arctic ecosystems are predisposed to face changes in the breeding phenology of migratory birds (Etzelmuller et al., 2011; Overland et al., 2014). These regions are also known for short‐term shifts in weather conditions that, in the case of migratory birds, translate into stochastic variation in daily food intake (Hoye & Forchhammer, 2008; Kwon et al., 2019; Saalfeld et al., 2019). These aspects make migratory Arctic birds a useful model system for investigating the fitness considerations of climate‐driven prolongation of the breeding season and stochastic fluctuations in food gain.

Interestingly, even closely related species of migratory birds breeding in the Arctic display considerable variation in breeding strategies along the continuum from pure capital to income breeding (Drent & Daan, 1980). Female common eiders (*Somateria mollissima*) are capital breeders that store extensive lipid reserves before nesting and use their internal reserves to boost reproductive effort and cover the energetic costs of incubation (Drent & Daan, 1980; Waltho & Coulson, 2015). In contrast, female king eiders (*S. spectabilis*) adopt a breeding strategy closer to income breeding as they continue to forage during egg laying and incubation (Drent & Daan, 1980; Waltho & Coulson, 2015). The stochastic variation in food gain is expected to interact with the body condition of these two eider species prior to and during the breeding season. In capital breeding species, variance in daily food gain prior to breeding affects clutch size and the ability to incubate eggs but the stored reserves from this period buffer unpredictable changes in body condition (Lameris et al., 2018). However, in income breeders, unexpected drops in body condition due to unsuccessful foraging can extend the period of egg laying and lead to failure to hatch offspring (Eeva & Lehikoinen, 2010). These differences in breeding strategies in response to stochastic variation in food gain are expected to result in differences in mortality and fecundity.

Here, we present a theoretical model in which populations of income and capital breeding eiders are exposed to earlier spring onset and different levels of variation in daily food gain. The offspring of both capital and income breeders produced late in the season have decreased chances of recruitment, so the timing of hatching affects fitness (cf. Drent et al., 2003; Morrison et al., 2019). Capital breeders in our model can increase clutch size by postponing the onset of nesting to increase pre‐breeding foraging. This creates a life history trade‐off between the number of eggs produced and offspring quality. However, income breeders need to continuously forage to produce subsequent eggs in a clutch. Our work shows that the fitness response to climatic changes in spring phenology depends on the degree to which migratory birds use internal reserves during breeding.

## 2 MATERIAL AND METHODS

### 2.1 Model overview

In this work, we present a life history optimization model (cf. McNamara & Houston, 2008) that represents the foraging and breeding strategies of migratory birds that differ in the degree to which reserves amassed prior to breeding are used during egg laying and incubation. A baseline version of the model was previously used to investigate the climate‐induced changes in breeding synchrony of common eiders under deterministic daily food intake (Ejsmond et al., 2021). The model presented here investigates fitness of income and capital breeders, approximated by the number of produced recruits, under stochastically fluctuating food gain. In the model, income and capital breeders arrive at breeding grounds with a lean body condition and the environmental conditions limit the start of nesting. The degree of stochastic fluctuations in daily food gain interacts with the time available for nesting constrained by the duration of nesting season. In capital breeders, females that forage longer before nesting are able to produce more eggs, but this leaves less time for the offspring to develop. However, income breeders need to continue foraging throughout egg laying, and the length of the nesting period limits the maximal number of eggs that can be laid. In both capital and income breeders, postponing nesting decreases the per‐offspring contribution to fitness as the timing of hatching affects the probability of recruitment.

The theoretical approach used here is a classic framework in life history evolution based on dynamic state variable modeling and the dynamic programming method (see details below). We used our model to determine the life history strategies that maximize fitness by running optimization backward in time (Houston & McNamara, 1999). The term “strategy” is used to indicate the physiological and behavioral responses that have evolved through natural selection. In our work, we first calculated the optimal, state‐dependent life history strategy in response to input parameters that defined the degree of stochastic fluctuation of daily food gain and seasonal time constraints. Next, we simulated a population of individual females that followed the optimal, state‐dependent strategy obtained with dynamic optimization. Both stages of the modeling, that is, the backward optimization and forward simulation are described in detail below. Although the parameters of our model were set to resemble the biology of eiders breeding in high‐latitude Arctic ecosystems, the conclusions from our work are relevant for any income and capital breeding migratory birds that produce one clutch per year.

### 2.2 Model inspiration

The model design was inspired by the breeding behavior of two species of sea ducks, the common eider and king eider, which nest in high‐latitude seasonal environments. These two species differ in the degree to which reproductive effort during the nesting period and incubation is sustained by reserves stored prior to breeding. While common eiders are considered to store extensive reserves prior to breeding, king eiders reportedly forage throughout egg laying and nesting (Waltho & Coulson, 2015). Thus, we modeled two strategies: an income breeder that needs to continuously forage to produce and lay eggs, and a capital breeder that uses reserves amassed prior to breeding to produce eggs and cover the costs of incubation. Below, we refer to the period of pre‐breeding foraging as the pre‐breeding season.

In the model, females arrived in spring at the breeding grounds in a lean condition (Milne, 1976; Waltho & Coulson, 2015). Correspondingly, the reserve level in arriving model females (*S*_min_) was set to 0. The total body mass of the modeled bird at day *t* consisted of the lean body mass *M*_L_ plus reserves *S*(*t*). The mass of the reserves increased as an effect of foraging, but the daily food gain in our model fluctuated stochastically. The maximal body mass (*M*_max_ = the sum of lean body mass *M*_L_ and maximal reserves *S*_max_) of the modeled birds could reach up to 130% of the lean body mass, which is consistent with empirical observations (Milne, 1976). Maximal clutch size *n*_max_ = 7 was parameterized to encompass interspecific variation in the clutch size of both species (Erikstad et al., 1993; Waltho & Coulson, 2015) with a laying rate of one egg per day (Watson et al., 1993). We further assumed that females produce eggs of the same size *E* = 110 g (Parker & Holm, 1990; Waltho & Coulson, 2015) by allocating *E* grams of energetic reserves and that they incubate the clutch for *I* = 26 days (Parker & Holm, 1990). To cover incubation costs, a female utilized reserves equal to 50% of the energetic costs needed to produce the eggs (cnE), with *c* set at 1.5 in our study.

The key parameter in our model was the first possible breeding day *b*_s_, that is, the day on which spring started. The timing of hatching determined the probability of offspring recruitment *f*(*t*), which was maximal at the first possible breeding day *b*_s_ of the earliest considered breeding season (among all investigated scenarios) and decreased linearly to 0, which marked the date at which it was too late for a duckling to develop before the autumn migration at time *T*. Decreasing probability of recruitment with the timing of hatching has been documented in several bird species (Drent & Daan, 1980; Morrison et al., 2019).

### 2.3 Stochastic foraging

Another key element of our model was the short‐term temporal variation in resource availability modeled by stochastically fluctuating daily food gain, *w*_*i*_. The daily change in energy reserves of foraging individuals is given by *S* (*t* + 1) *= S* (*t*) + *w*_*i*_, with *w*_*i*_ equal to the net outcome of foraging, including the energetic costs of searching, prey handling, etc. Hence, net daily food gain *w*_*i*_ could turn negative if the modeled bird was unable to cover the costs of metabolism, locomotion, maintenance, etc. We further assumed that the reserves could not turn negative, so if *S* (*t*) + *w*_*i*_ < 0, then *S* (*t* + 1) was set to 0. The average net daily food gain for model eiders, *w*_a_, was set to 15 g per day, which is consistent with the empirical estimate (Korschgen, 1977; Rigou & Guillemette, 2010). Due to the stochastic nature of the foraging process, the daily net portion of acquired resources in our model varied between *x*_1_ *w*_a_ and *x*_2_ *w*_a_. Below, we present results for *x*_1_ = −1 and *x*_2_ = 3, which indicates that an individual unsuccessful in obtaining food could lose a mass of reserves equivalent to average daily food gain *w*_a_ and a successful individual that had a maximal net resource acquisition three times higher than *w*_a_. The conclusions of our work did not change for a broad range of tested values of average food gain *w*_a_ and of the parameters *x*_1_ and *x*_2_ that set the stochastic distribution of the daily food gain *w*_*i*_.

**FIGURE 1:**
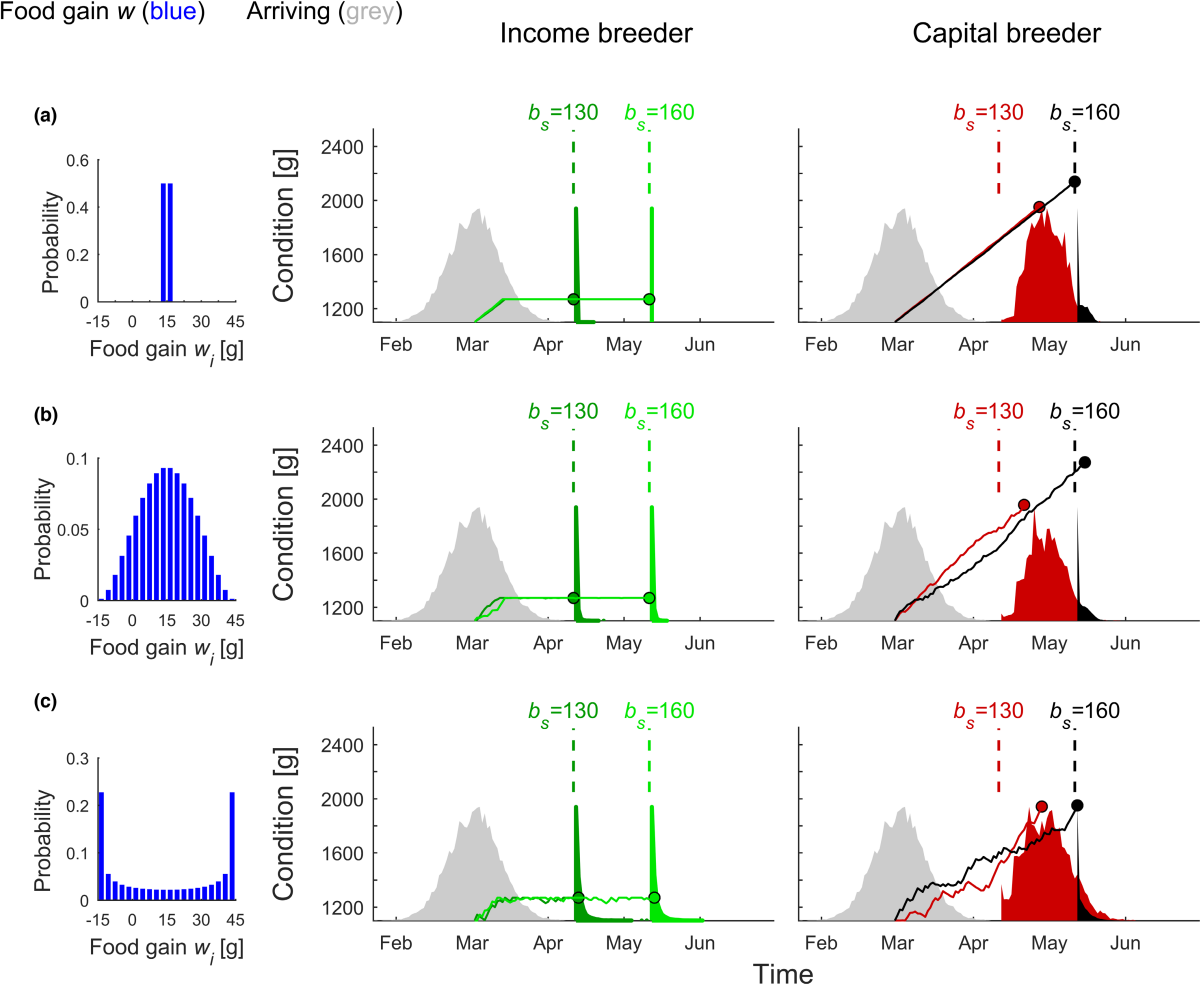
Optimal breeding strategies for three scenarios of fluctuating net food gain with example trajectories of females' body condition. Individual female income breeders (panels, middle column) and capital breeders (panels, right column) optimize the timing of breeding in response to the assumed distribution of net daily food gain *w*_*i*_ (panels, left column). The optimal strategies of females are presented for two scenarios differing in the time of first possible nesting *b*_*s*_ (see labels of dashed lines in the middle and right columns of panels). Trajectories show example changes in body conditions of females exposed to fluctuating net food gain (blue). The timing of arrival for the modeled population of 10,000 females (gray) was sampled from the normal distribution with mean arrival date *D*_a_ = 90 and standard deviation SD = 10. The stochastic fluctuations of daily net food gain were given by the *β* probability distribution with shape parameters (a) *α* = 500, (b) *α* = 3, and (c) *α* = 0.5. Maximal and minimal net daily food gain were set to *x*_1_ *w*_a_ = −1 and *x*_2_ *w*_a_ = 3 (see Section 2: Materials and methods).

Each day of foraging resulted in a specific outcome in terms of net food gain. We assumed that there were *i* = 1, 2, … *k* foraging outcomes in our model that each had the probability *p*_*i*_ of occurring and resulted in daily net food gain of *w*_*i*_. In the results presented below, we assumed *k* = 20. Changing *k* to a higher value, that is, considering more foraging outcome types, did not affect the result of the model but prolonged the calculation times. The probabilities *p*_*i*_ were calculated from a β distribution. We used a β distribution because it is defined on the interval [0, 1], which, after rescaling, allowed us to keep the food gain within a predefined interval of *x*_1_ *w*_a_ and *x*_2_ *w*_a_. We also assumed that the probability distribution of daily food gain was symmetric, that is, the shape parameters *α* and *β* of the distribution were kept equal. By setting the *α* shape parameter of the distribution, we were able to explore a broad range of scenarios differing in the variance of but not the average net daily food gain set to *w*_a_ in our model (see the left column panel in Figure 1).

### 2.4 Backward optimization

We used the backward optimization procedure to determine the optimal reproductive strategy with maximal fitness approximated by the expected number of produced recruits *V* at time *T*, that is, at the end of the season according to:

(1) $$V\left(T\right)=\mathrm{nf}\;\left(t_{c}\right)$$

with the number of eggs *n* laid by the female and the recruitment probability *f* at the time of hatching *t*_c_. However, capital and income breeders differed in their optimal breeding strategy and how fitness *V* (*S*, *n*, *t*) was calculated. The technique used finds the expected fitness pay‐offs assigned to actions taken on a given day *t* in a season by a modeled female bird characterized by a certain reserve level, *S*, and a certain number of eggs laid, $n\in \left\{1,2,\ldots ,n_{\max}\right\}$ (see Ejsmond et al., 2021).

A capital breeder that started incubating was assigned fitness $V_{\text{incubate}}$ that depended on *n*, the number of eggs laid, and *f*, the chance of offspring recruitment after hatching, which declined with time

(2) $$V_{\text{incubate}}\left(t,n\right)=n\;f\;\left(t+i\right),\text{for}\;b_{s}\leq t\leq T-I\;$$

where the number of eggs laid could not exceed *S*(*t*)/(cE), the clutch was incubated for *I* days, and hatching took place *i* days after the last egg was laid. A female that produced fewer eggs than *n*_max_ could continue egg laying, which would lead to the fitness $V_{\mathrm{lay}}$ calculated as follows

(3) $$V_{\mathrm{lay}}\left(S,t,n\right)=V\left(S-\mathrm{cE},\;t+1,\;sn+1\right),\text{for}\;S\geq \mathrm{cE}$$

The female could also continue foraging, which would influence prospects for future reproduction by increasing her reserves, *S*. The expected increase in reserves (and thus the fitness *V*_forage_) was calculated as follows

(4) $$V_{\text{forage}}\left(S,t,n=0\right)=\sum_{i=1}^{k}p_{i}V\left(S+w_{i},\;t+1,\;n=0\right)$$

where *w*_*i*_ is the daily food intake of type *i* that could occur with probability *p*_*i*_. The third argument (*n* = 0) indicates that for capital breeders, foraging terminated when the first egg was laid. Capital breeders could not store more reserves than $S_{\max}$. The model compares and extracts the maximal value from $V_{\text{forage}}$, $V_{\mathrm{lay}},$ and $V_{\text{incubate}}$ calculated on each day *t* for each level of energy reserves *S* and number of eggs laid *n* as follows

(5) $$V\left(S,t,n\right)=\left\{\begin{array}{c} \max\left[V_{\text{forage}}\left(S,t,n\right),V_{\mathrm{lay}}\left(S,t,n\right),V_{\text{incubate}}\left(t,n\right)\right],\text{for}\;n=0\\ \max\left[V_{\mathrm{lay}}\left(S,t,n\right),V_{\text{incubate}}\left(t,n\right)\right],\text{for}\;0<n<n_{\max}\\ V_{\text{incubate}}\left(t,n\right),\text{for}\;n=n_{\max} \end{array}\right.$$

Thus, the model optimized the day t* at which a capital breeding female initiated egg laying by switching from foraging to reproduction and the day t** at which the female terminated egg laying and started incubation, which determined the clutch size.

In income breeders, the maximal allowed reserves were equal to the production cost of one egg, that is, *S*_max_ = cE. Similar to capital breeders, income breeders were exposed to stochastically varying food gain, and foraged and laid eggs until time t** when the 26‐day incubation period started. In income breeders, the onset of incubation at t** determined clutch size. Income breeders were allowed to forage and increase their reserves *S* regardless of the number of eggs already laid (cf. Equation (4)) as follows:

(6) $$V_{\text{forage}}\left(S,t,n\right)=\sum_{i=1}^{k}p_{i}V\left(S+w_{i},t+1,n\right)$$

The state‐dependent reproductive strategy that maximized the fitness of income breeders was determined using dynamic optimization according to

(7) VS,t,n=maxVforageS,t,n,VlayS,t,n,Vincubatet,n,forn<nmaxVincubatet,n,forn=nmax

The reserves level was a continuous variable in our model, but reproductive value was calculated for discrete values. We use linear interpolation for discretization (Clark & Mangel, 2000) and the same interpolation was used in the forward simulations (see below).

### 2.5 Forward simulation

We simulated the behavior of 10,000 females with normally distributed arrival dates to calculate average nesting onset, defined as the date of laying the first egg, clutch size, and the number of recruits produced by populations of income or capital breeders. The behavior of the simulated females followed the state‐dependent optimal strategy t* and t** calculated with backward optimization procedure (see above and Equation (5)) for various assumed probability distributions of daily food gain *w*_*i*_ and dates of the first possible day of nesting onset *b*_s_ (Figure 1). In forward simulations, the food gain at any given day was drawn from the probability distribution used to obtain state‐dependent optimal strategy t* and t**. The assumed migration pattern of the modeled population of eiders (mean arrival date *D*_a_ = 90, SD = 10 days) was consistent with empirical data on the migration of high‐latitude populations of common eiders breeding in Svalbard (Ejsmond et al., 2021; Hanssen et al., 2016; Waltho & Coulson, 2015). The assumed distribution of arrival dates introduced variation in body condition of females. However, the conclusion derived from our work did not change as long as this variation in body condition of females was maintained, for example, by assuming that females differ with their body condition at arrival. For a detailed sensitivity analysis of the model to key parameters, see Ejsmond et al. (2021). All calculations were performed with MATLAB (R2015b).

## 3 RESULTS

Our model compared the breeding behavior and fitness components for income and capital breeders exposed to varying degrees of fluctuations in the net daily food gain *w*_*i*_ and first possible day of nesting onset *b*_s_. In scenarios with fluctuating resource availability, birds gained the same amount of food on average as in a deterministic environment, but the fluctuations introduced variation in the body condition of individuals, as some reached a certain level of reserves faster or slower than the population average (compare trajectories in Figure 1c with those in 1a). Because the optimal strategies of both income and capital breeders strongly depended on time constraints related to the duration of the pre‐breeding and breeding seasons, the fluctuating food gain interacted with the date of spring onset, specified by *b*_*s*_ in our model (Figure 1). To explain the observed changes in the fitness of income and capital breeders in the modeled scenarios, we first describe the breeding phenology of the modeled strategies.

### 3.1 Income breeders

**FIGURE 2:**
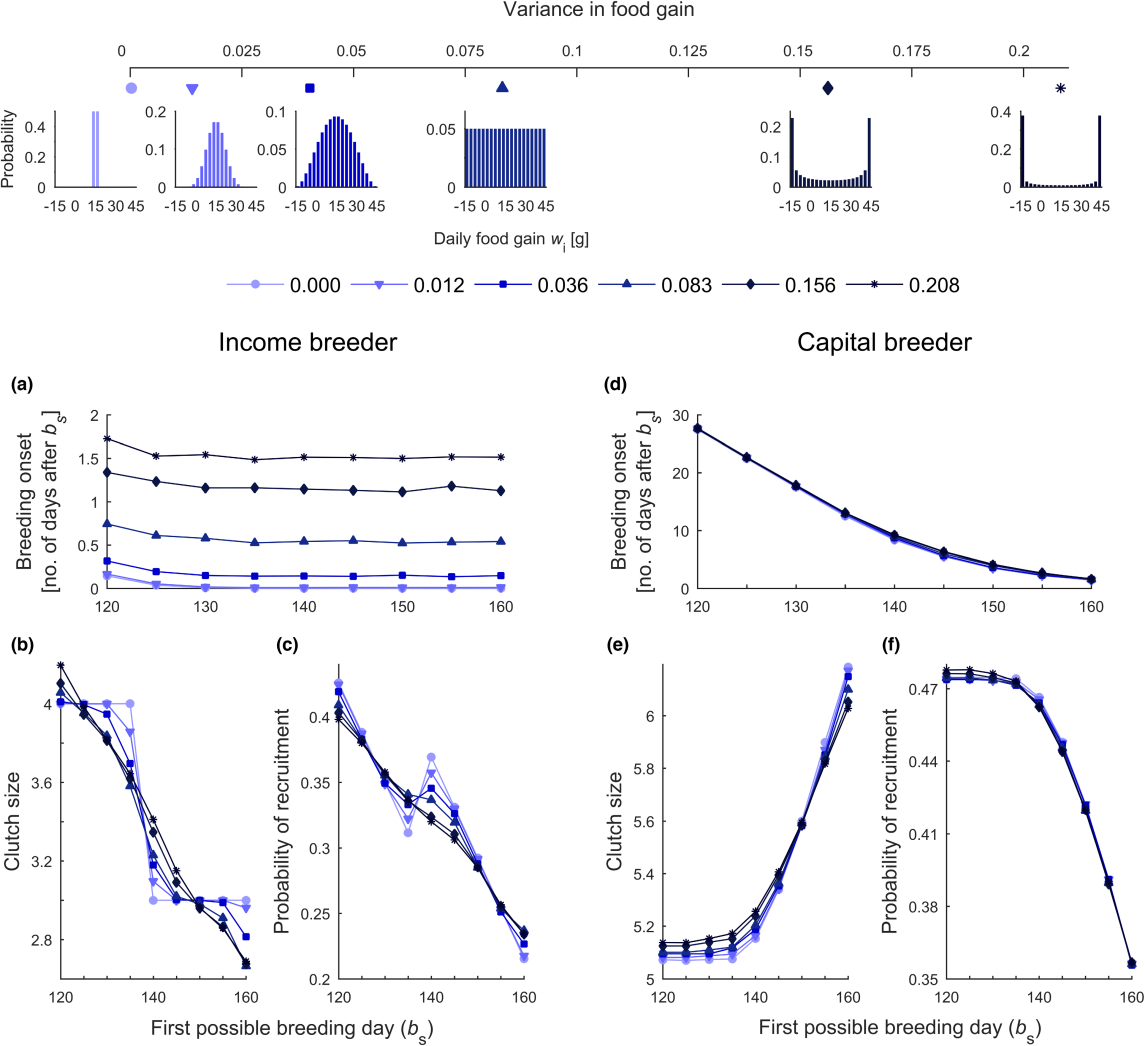
Breeding onset, clutch size, and probability of recruitment in the modeled populations. The top panel presents distributions of net daily food gain *w*_i_ that differ in variance (see the top axis and legend); the colors and shapes match the scenarios presented in A‐F. (a, d) Average timing of nesting relative to the first possible breeding day *b*_*s*_, (b, e) average clutch size, and (c, f) probability of offspring recruitment. (a–f) Low values of *b*_s_ represent scenarios with early spring onset for income and capital breeders (see labels at the top of the panels).

**FIGURE 3:**
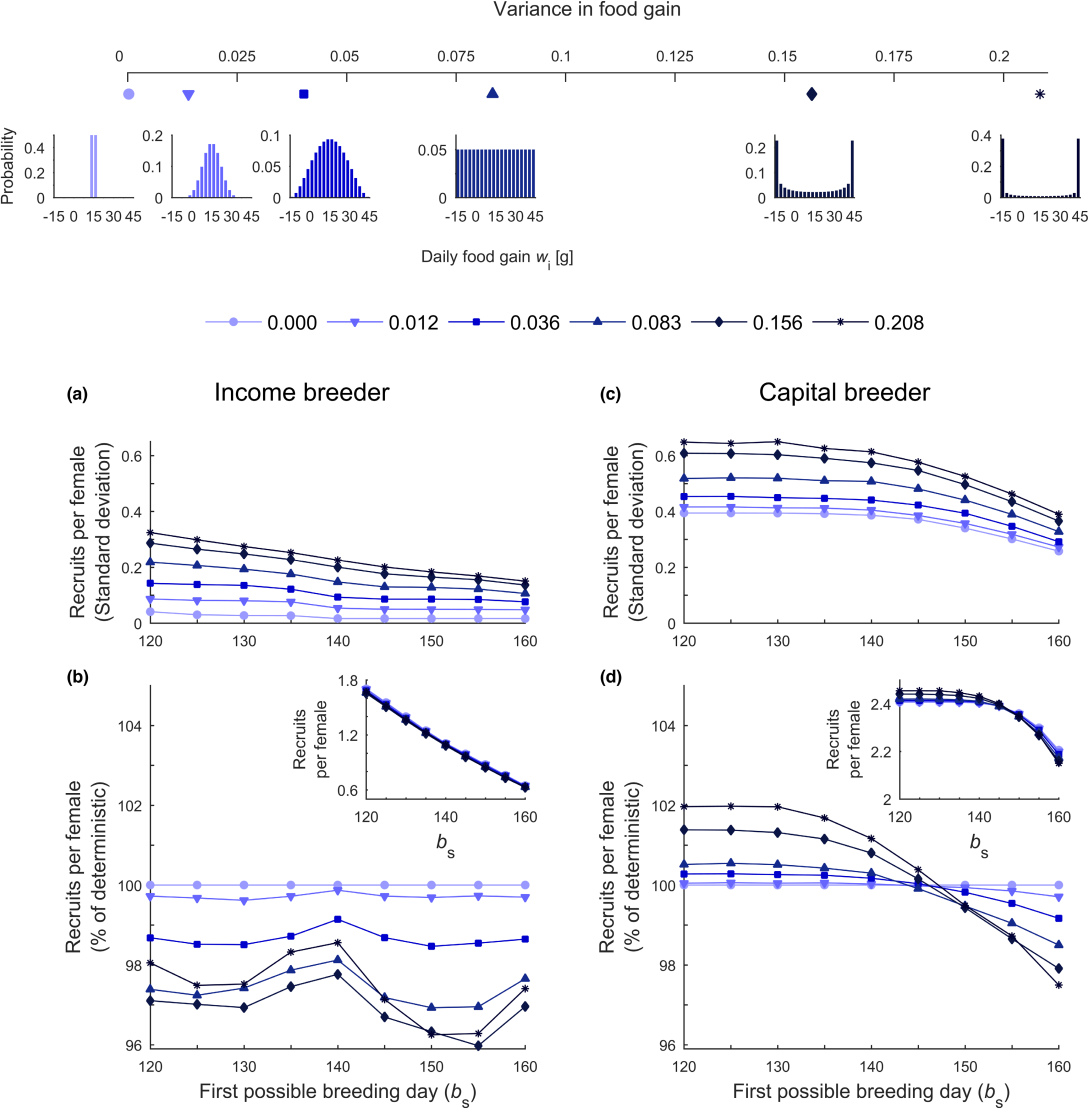
Average number and variance in recruitment. The top panel presents distributions of net daily food gain *w*_i_ that differ in variance (see the top axis and legend); the colors and shapes match the scenarios in (a–d). (a, c) Intrapopulation variation in recruitment and (b, d) average number of recruits per female (given in insets) expressed relative to the recruitment under deterministic food gain. (a–d) Low values of *b*_s_ represent scenarios with early spring onset, and measures of recruitment are given for populations of income and capital breeders (see labels at the top of the panels).

Postponing the timing of nesting did not benefit the fitness of income breeders once females had amassed sufficient reserves to produce an egg. In a deterministic environment, income breeders started nesting immediately after the first possible breeding day *b*_s_ (Figure 2a, light blue line). The only situation that delayed nesting onset by income breeders was a very early spring onset in which some late‐arriving birds needed to forage beyond *b*_s_ to gather resources for their first egg (Figure 2a). Under conditions of fluctuating resource gain, greater variance in food gain generated greater delay of nesting onset for income breeders (Figure 2a), as due to fluctuations, some birds needed more time to amass reserves for the first egg than the population average. Regardless of the fluctuations in food gain or spring onset, income breeders capable of laying an egg would decrease their fitness by postponing nesting onset. Hence, income breeders produced large clutches only with early spring onsets (Figure 2b). However, early spring onset allowed not only the production of large clutches (Figure 2b) but also an earlier start of incubation and, in turn, a high probability of recruitment (Figure 2c). In deterministic environments, the maximal fitness was reached at a certain combination of clutch size and value of offspring at hatching (Figure 2b,c). Note that nearly all income breeders in the deterministic environment applied the same breeding strategy, as the variation in the number of recruits per female was close to zero (Figure 3a, see the light blue line). However, in stochastic environments, income breeders produced, on average, smaller clutches with higher per‐offspring value or larger clutches with smaller per‐offspring value (Figure 2b,c). The greater the variance in food gain, the greater the variation in the fitness of income breeders (Figure 3a). Hence, the number of recruits per female dropped as the degree of stochastic fluctuations in food gain increased (Figure 3b).

### 3.2 Capital breeders

The timing of nesting of capital breeders was more complex. Capital breeders, in contrast to income breeders, were able to increase reserves and produce larger clutches by postponing reproduction and continuing to forage prior to nesting. The onset of nesting in capital breeders depended on the date of spring onset: the earlier the *b*_s_ was, the greater the relative delay in nesting (Figure 2d). This delay in nesting onset was further increased by fluctuations in food gain (Figure 2d).

In contrast to income breeders, capital breeders produced smaller clutches in years with early spring onsets (Figure 2b vs. 2e) which was associated with a high probability of offspring recruitment (Figure 2f). Capital breeders in good condition started egg laying immediately or just after *b*_s_, whereas those in poor condition continued foraging after spring onset, which translated into higher variation in the number of produced recruits (Figure 3a vs. 3c). Under conditions of fluctuating food gain, the variation in the number of recruits increased (Figure 3c). However, in scenarios with early spring onset and stochastically fluctuating food gain, capital breeders, but not income breeders, were able to produce more recruits per female on average than in scenarios with deterministic food gain (Figure 3b vs. 3d). This positive effect of fluctuating food gain on the fitness of capital breeders was present only in scenarios with early spring onset (Figure 3d).

## 4 DISCUSSION

The onset of spring and the nesting of many migratory bird species have shifted toward earlier dates, relaxing the temporal constraints on offspring development (Drent et al., 2003; Morrison et al., 2019; Van Der Jeugd et al., 2009). Given the advancing onset of breeding by various taxa worldwide, stochastic variation in resource availability is expected to play a significant role in shaping the reproductive success of migratory species (Forchhammer & Post, 2000; Hoye & Forchhammer, 2008; Kwon et al., 2019; Visser & Gienapp, 2019). However, it is unclear how increased stochastic fluctuations in food availability will affect the reproductive success of migratory birds that nest earlier in warming environments. In our model, predictions regarding the fitness consequences of climate‐induced shifts in nesting phenology and temporal variation in food availability depended on the adopted breeding strategy (cf. Jönsson, 1997). In years with earlier spring onset, capital breeders coped better than income breeders with fluctuations in food gain. Importantly, the average number of recruits produced by capital breeding birds with earlier nesting onset increased with the degree of stochastic fluctuations in food gain, reaching levels above those predicted in scenarios with constant food. This was because individuals that frequently experienced high food gain were able to produce large clutches at or shortly after spring onset, which contributed to the high average clutch size (Figure 3c). Individuals that frequently experienced low food gain were still able to forage until they amassed sufficient reserves to produce a clutch. When spring onsets were late, a large proportion of arriving capital breeders were able to gather the maximal possible reserves before *b*_s_. In these scenarios, the strong fluctuations in food gain decreased an individual's condition every time they experienced negative net food gain, and consequently, the average fitness was lower than under deterministic environments. Hence, internal reserves for reproduction buffered the effect of uncertainty in food gain and allowed birds to attain higher fitness on average than that under constant food gain. Income breeders were unable to buffer this uncertainty, and the number of recruits per female always dropped as the degree of stochastic fluctuations in food intake increased, no matter the date of spring onset.

Our work has important implications for predicting the fitness response of migratory birds that produce one clutch per season to climate‐induced environmental change. The positive fitness consequences of capital breeding are often correlated with large structural body size of species (Ejsmond et al., 2018; Trillmich & Weissing, 2006; Walczyńska et al., 2010). We predict that the physiological adaptation for storing internal reserves prior to breeding in large‐bodied migratory species among ducks, geese, swans, gulls, birds of prey, etc. would allow them to buffer fitness loss or even increase recruitment in years with earlier spring onsets and higher temporal variation in food gain (Ejsmond et al., 2021; Lameris et al., 2018). Regardless of the potential shifts in spring onset, pronounced temporal variation in food availability is expected to always reduce the recruitment of small‐bodied passerines that are restricted in the amount of internal reserves by the constraints on flight ability and consequently increased mortality risk (e.g., Kullberg et al., 2005).

Climate change has shifted spring onset earlier worldwide and introduced local disturbances due to increased spatiotemporal stochastic variation in food resources (Assmann et al., 2019; Forchhammer & Post, 2000; Kwon et al., 2019). The retreat of sea ice and warming of high‐latitude marine ecosystems are forecasted to locally decrease biomass of the primary food resource of eiders—populations of blue mussels. These drops in biomass are expected due to a decline in the quality of organic matter settling out of the water column (Kedra et al., 2015) or warming winters that force mussels to remain active during periods with negative energy balance (Waldeck & Larsson, 2013). In many geographic regions, blue mussel beds have non‐uniform and complex spatial structure over a relatively small geographic scale with population density ranging from 0 to packs of mussels overgrowing each other (Commito et al., 2014). The inflow of warmer and less saline waters, both outcomes of climate change, may lead to local drops in population density or even local extinctions of blue mussels (Jaatinen et al., 2021). Blue mussels change shape of their shells, that is, a primary determinant of the ratio of soft tissues to shell mass, in response to inflow of water with increased temperature and low salinity (Telesca et al., 2018). The climate‐induced changes mentioned above are likely to concern local populations of blue mussels predicted and increase spatiotemporal variation in the quality of eider foraging patches.

The random nature of foraging is an important driver of the optimal behavior and life history strategies adopted by animals (Fischer et al., 2009, 2011; Stephens & Charnov, 1982). In eiders, the stochastic foraging elements of finding an optimal foraging patch and searching for prey play an important role, as these sea ducks dive to feed on organisms living on the sea bottom. Diving for benthic organisms consumes significant amounts of time and energy, and eiders feeding on small organisms need to collect several prey items to counter the costs of searching for food (Waltho & Coulson, 2015). In poor‐quality areas along the seacoast or under high spatiotemporal variation in food availability, the costs of searching for food can outweigh the energetic gains from foraging (Camphuysen et al., 2002). Climate‐induced changes in food availability interact with foraging behaviors that have stochastic outcomes. Potential changes in weather conditions are also expected to affect the efficiency of prey handling and searching for optimal foraging patches. These outcomes are expected to magnify the non‐deterministic nature of food acquisition under climate‐induced changes in environmental conditions. According to our model, migratory species capable of storing extensive reserves prior to breeding are particularly predisposed to cope well with the climate‐induced increase in spatiotemporal variation in food gain given earlier spring onset.

The timing of nesting, clutch size, and recruitment of capital and income breeders under fluctuations in food gain were modeled in individuals that differed in the timing of spring migration and arrived in lean body conditions. In our model, pre‐breeding foraging introduces variance in body conditions at spring onset, but in natural conditions, numerous forces can affect the body conditions of females upon arrival. Since we focused on individual body conditions at the time when environmental conditions allow nesting, the conclusions of our work also hold when birds arrive on the same day but differ in body condition upon arrival. Our model is applicable to migratory as well as non‐migratory birds that produce one clutch per year, as long as they exhibit pre‐breeding variation in body condition before the environmental conditions allow nesting (Lameris et al., 2018; Rowe et al., 1994).

According to the results, in years with early spring onset, capital breeders produce smaller clutches (cf. Ejsmond et al., 2021), whereas common eider females in the Baltic Sea population lay more eggs when spring arrives early (Öst et al., 2022). In our model, early spring onset shortened pre‐breeding period, but body condition of arriving females was not correlated with timing of spring onset. These assumptions imply a weak correlation between climatic conditions at wintering and breeding grounds, which likely fit the breeding strategies of high Arctic population of common eiders overwintering in Iceland and Northern Europe (Ejsmond et al., 2021; Hanssen et al., 2016). However, in the case of the Baltic Sea population, wintering and breeding grounds are relatively nearby, and it is more likely that in years with early spring onset females arrive at breeding grounds in better body condition. This could explain production of larger clutches in years with early spring onset observed in the Baltic Sea population, or predicted by the theoretical models that link the timing of spring arrival with body condition of migrating females (Descamps et al., 2011).

Our theoretical life history approach shows that capital breeding allows attaining higher fitness by migratory birds exposed to pronounced fluctuations of food gain. The revealed fitness response to the interaction between the temporal constraints of reproductive success, manifested by advanced spring onset, and stochastic variation in the environment was derived from the state‐dependent resource allocation. To conclude, the degree to which migratory bird species utilize energetic reserves during reproduction influences their fitness response to climate‐induced changes in the onset of nesting season.

## AUTHOR CONTRIBUTIONS

**Anna Ejsmond:** Conceptualization (lead); formal analysis (lead); funding acquisition (lead); investigation (equal); methodology (equal); project administration (lead); software (lead); validation (equal); visualization (equal); writing – original draft (lead); writing – review and editing (equal). **Maciej Jan Ejsmond:** Conceptualization (equal); formal analysis (supporting); investigation (supporting); methodology (equal); project administration (supporting); software (supporting); supervision (lead); validation (equal); visualization (equal); writing – original draft (supporting); writing – review and editing (equal).

## FUNDING INFORMATION

University of Bergen funds and University of Iceland Research Fund, Grant number: 2900–293501.

## CONFLICT OF INTEREST

The authors do not have any conflicts of interest to declare.

## Supporting information


Appendix S1

## Data Availability

The computer program used in the work is available in the Supplementary Information (see Appendix S1).
