# Supplementary material for: Food resource uncertainty shapes the fitness consequences of early spring onset in capital and income breeding migratory birds
Source: Ecol Evol. 2022 Dec 21;12(12):e9637. doi: 10.1002/ece3.9637 (PMC9771707; doi:10.1002/ece3.9637)
Supplement: Supplementary file 1 — Appendix S1 [file ECE3-12-e9637-s001.pdf]

```
clear; clc; close all; tic
% The model consist of two parts: backward optimization of optimal breeding
strategy and forward simulations of arriving females
% The model optimizes timing of breeding, clutch size and derives breeding
synchrony
% ##########################################################################
%Set PureCap==1 to model Capital breeder or set PureCap==0 to model Income breeder
PureCap=1;
% ##########################################################################

%% PARAMETERS AND SETTINGS
%Food gain distribution - 3 scenarios
AlfBet=[500 .3]; %alfa and beta in the Beta distribution are kept equal
Prebreeding_weight_gain = 15;  %weight gain
wm=-1; %minimal food gain - fraction of the baseline daily food gain w (wm can be
negative)
wx=abs(wm)+2; %maximal food gain - fraction of baseline daily food gain w

Day_start=130; %Day start
Day_end = 240; %Last day of possible egg laying [day of year]
Clutchsize_max = 6; % maximal clutch size
Incubation = 26; %days
Day_max = ceil(Day_end+Clutchsize_max+Incubation);%last possible hatching day
Eggweight = 110; %grams
Eggproductioncost = 1.5; %Factor accounting for all extra costs of egg production
(assume that this includes incubation cost)
LeanBodyMass = 1100; %grams
MaxBodyMass = 2500; %grams
Reserves_max = MaxBodyMass - LeanBodyMass;%grams
Condition_maxcat = 150; %resolution of grid for backward optimization
Mean_arriv1=90; %mean arival date
SD1=10; %standard devition
N=10000; %population size for forward simulations

%Prelocation of variables used to gather history of individuals in forward
simulation:
Arriv_popul=zeros(size(AlfBet,2),N);
Hatch_popul=zeros(size(AlfBet,2),N);
Clutchsize_popul=zeros(size(AlfBet,2),N);
Offval_popul=zeros(size(AlfBet,2),N);
ClutchSizAv=zeros(size(AlfBet,2),1);
ProbRecrAv=zeros(size(AlfBet,2),1);
RecrNoAv=zeros(size(AlfBet,2),1);
RecrSD=zeros(size(AlfBet,2),1);
StandBinCentr = (0:0.05:1-0.05)+(0.05/2);%normalization of bins
BinEdeEndingBin = StandBinCentr + (0.05/2);

% scenarios:
zapis_FeedDistr=zeros(size(AlfBet,2),length(BinEdeEndingBin),2);

for cmb_i2=1:size(AlfBet,2)
    disp(['Alpha=',num2str(AlfBet(cmb_i2)),'    Beta=',num2str(AlfBet(cmb_i2))])
    %STOCHASTIC FOOD GAIN
```

```
    alfbet=AlfBet(cmb_i2);
    Prob_DstGain=diff(betacdf([0 BinEdeEndingBin],alfbet,alfbet));%Probability↙
that a given level of daily food gain happens
    FodGaIn=abs(wm-wx)/length(Prob_DstGain);%defining bins
    Val_DstGain=wm:FodGaIn:wx; %Fraction of the baselien food gain w (defined↙
below), e.g. Val_DstGain=[-0.2 1 1.2] match -20% 100% and 120% of w
    Val_DstGain=Val_DstGain(1:end-1)+(FodGaIn/2);%bin centers calculated - certain↙
levels of food gain can happen with probability given in Prob_DstGain
    Vect_wghtGain = Val_DstGain .* Prebreeding_weight_gain;
    lStG=length(Val_DstGain);
    zapis_FeedDistr(cmb_i2,:,1)=Prob_DstGain;
    zapis_FeedDistr(cmb_i2,:,2)=Val_DstGain;

    %Initialize offspring fitness as function of day of hatch
    offvalue=zeros(1,Day_max);
    DayAtOfVal_1=min([Day_start 110]);
    for Day = DayAtOfVal_1:Day_end
        offvalue(Day) = 1-(Day-DayAtOfVal_1)/(Day_end-DayAtOfVal_1);
    end

    %% BACKWARDS OPTIMIZATION
    %Prelocation of Strategy and fitness matrix
    Fitness=zeros(Condition_maxcat,Day_max,Clutchsize_max+1);
    Strategy=ones(Condition_maxcat,Day_max,Clutchsize_max+1);
    for Day = Day_end:-1:Day_start
        for Condition = 1:Condition_maxcat
            Reserves = (Condition-1)/(Condition_maxcat-1) * Reserves_max;%grams
            for EggsInNest = 0:Clutchsize_max
                idxEgInNe=find(EggsInNest==(0:Clutchsize_max));
                %FORAGING - ACTION (1)
                FitnessIfForagingVect=zeros(1,lStG);
                for ilst=1:lStG
                    PrebWgGain_i=Vect_wghtGain(ilst);
                    if PureCap==1 %CAPITAL BREEDER
                        if EggsInNest==0
                            Reserves_new = Reserves +  PrebWgGain_i;
                            if Reserves_new<0
                                Reserves_new=0;
                            end
                        else
                            Reserves_new = Reserves;
                        end
                    else %INCOME BREEDER
                            Reserves_new = min([Reserves+PrebWgGain_↙
(Eggweight*Eggproductioncost)*1.1]);
                        if Reserves_new<0
                            Reserves_new=0;
                        end
                    end
                    %for interpolation
                    Condition_new = (Reserves_new/Reserves_max)*(Condition_maxcat↙
1)+1;  %decimal value
                    intC = max(1, min(floor(Condition_new),Condition_maxcat-1)); %↙
```

```
integer part
                    dC = max(0., min(Condition_new-intC , 1));  %decimal part
                    FitnessIfForagingVect(ilst) = dC * Fitness(intC+1, Day+1↙
idxEgInNe) + (1-dC) * Fitness(intC, Day+1, idxEgInNe);
                end
                FitnessIfForaging = sum(FitnessIfForagingVect.*Prob_DstGain);
                %LAYING ONE EGG - ACTION 2
                if (Reserves>=(Eggweight*Eggproductioncost) &&↙
EggsInNest<Clutchsize_max)
                    Reserves_new_lay = Reserves - (Eggweight*Eggproductioncost);
                    %for interpolation
                    Condition_new_Lay = (Reserves_new_lay/Reserves_max)↙
(Condition_maxcat-1)+1; %decimal value
                    intC = max(1, min(floor(Condition_new_Lay),Condition_maxcat↙
1)); %integer part
                    dC = max(0., min(Condition_new_Lay-intC , 1));%decimal part
                    FitnessIfLayEgg = dC * Fitness(intC+1, Day+1, idxEgInNe+1) ↙
(1-dC) * Fitness(intC, Day+1, idxEgInNe+1);
                else
                    FitnessIfLayEgg=0;
                end
                %INCUBATION - ACTION 3
                if (Day+Incubation)<Day_end
                    FitnessIfIncubate=EggsInNest.*offvalue(Day+Incubation);
                else
                    FitnessIfIncubate=0;
                end
                %OPTIMIZATION - IF ALL FITNESS COMPONENTS EQUAL 0 THEN THE FEMALE↙
FORAGES
                FitCompo=[FitnessIfForaging FitnessIfLayEgg FitnessIfIncubate];
                mxFit=max(FitCompo);
                Act=find(mxFit==FitCompo,1,'first');
                Fitness(Condition,Day,idxEgInNe) = FitCompo(Act);
                Strategy(Condition,Day,idxEgInNe) = Act;

            end
        end
    end

    %% FORWARD SIMULATION
    arriving_pop1=round(normrnd(Mean_arriv1,SD1,1,N));%Arriving population
    csProb_DstGain=cumsum(Prob_DstGain);%drawing food gain in forward simulation

    %Prelocation of variable holding info about arriving females:
    vect_offval=zeros(size(arriving_pop1));%clutch value
    trj_Cond=NaN(length(arriving_pop1),Day_max);%trajectories of condition
    trj_egg=NaN(length(arriving_pop1),Day_max);%trajectories of egg laid
    for i = 1:length(arriving_pop1)
        trj_Cond(i,arriving_pop1(i)) = 0;%grams
        trj_egg(i,arriving_pop1(i)) = 0;%eggs in nest
        for Day = arriving_pop1(i):Day_end%loop stops at last possible day of egg↙
laying
            Reserves = trj_Cond(i,Day);
```

```
            EggInNest_sim=trj_egg(i,Day);
            if Reserves>Reserves_max
                Reserves=Reserves_max;
            end
            Condition = floor((Reserves/Reserves_max)*(Condition_maxcat-1)+1);
            idx_EgINe = find(EggInNest_sim==(0:Clutchsize_max));
            Action = Strategy(Condition,Day,idx_EgINe);
            if Action==1 %FORAGE
                StochWghtGain=Vect_wghtGain(sum(csProb_DstGain<rand(1))+1);
                if PureCap==1 %CAPITAL BREEDER
                    if EggInNest_sim==0
                        Reserves_new = Reserves + StochWghtGain;
                        if Reserves_new<0
                            Reserves_new=0;
                        end
                    else
                        Reserves_new = Reserves;
                    end
                    trj_Cond(i,Day+1) = Reserves_new;  %grams
                    trj_egg(i,Day+1) = EggInNest_sim;
                else %INCOME BREEDER
                    GridInter=((1:Condition_maxcat)-1)/(Condition_maxcat-1) ↙
Reserves_max;
                    ModifEggweight=GridInter(find(GridInter<↙
(Eggweight*Eggproductioncost),1,'last')+1);
                    Reserves_new = min([Reserves+StochWghtGain  ModifEggweight]);
                    if Reserves_new<0
                        Reserves_new=0;
                    end
                    trj_Cond(i,Day+1) = Reserves_new;  %grams
                    trj_egg(i,Day+1) = EggInNest_sim;
                end
            elseif Action==2 %LAY EGG
                if (Reserves>=(Eggweight*Eggproductioncost) &&↙
EggInNest_sim<Clutchsize_max)
                    Reserves_new = Reserves - (Eggweight*Eggproductioncost);
                    trj_Cond(i,Day+1) = Reserves_new;
                    trj_egg(i,Day+1) = EggInNest_sim+1;
                else
                    trj_Cond(i,Day+1) = Reserves;
                    trj_egg(i,Day+1) = EggInNest_sim;
                end
            elseif Action==3 %START INCUBATION
                vect_offval(i)=offvalue(Day+Incubation);
                break
            end
        end
    end
    trj_Cond=trj_Cond+LeanBodyMass;
    %Calculating data for distributions of arrival and breeding dates
    breed_date=trj_egg;
    breed_date(isnan(breed_date))=0;
    breed_date=breed_date==0;
```

```
    breed_date=sum(cumprod(breed_date,2),2)';
    vect_Clutchsize=max(trj_egg,[],2)';
    Arriv_popul(cmb_i2,:)=arriving_pop1;
    Hatch_popul(cmb_i2,:)=breed_date;
    Clutchsize_popul(cmb_i2,:)=vect_Clutchsize;
    Offval_popul(cmb_i2,:)=vect_offval;

    [distr_pop_arriving,~]=histcounts(arriving_pop1,0:Day_max);%distribution of↙
arrival dates
    [distr_pop_breeding,~]=histcounts(breed_date,0:Day_max);%distribution of↙
breeding initiation dates
    [distr_pop_clutchsize,~]=histcounts(vect_Clutchsize,1-0.5:Clutchsize_max+0.5)↙
%distribution of clutch size
    [distr_pop_offval,~]=histcounts(vect_offval,0:0.1:1.1);%distribution of clutch↙
value
    [distr_pop_recr,~]=histcounts(vect_Clutchsize.*vect_offval,0:0.1:4);%↙
distribution of recruitment per female
    eval(['zapis',num2str(cmb_i2),'_trj_Cond=trj_Cond;']);
    eval(['zapis',num2str(cmb_i2),'_trj_egg=trj_egg;']);
end
zapis_Mean_arriv1=Mean_arriv1;
zapis_SD1=SD1;
zapis_N=N;
zapis_Day_max=Day_max;
etyk_save=['DayS',num2str(Day_start),'_FodGn',num2str↙
(Prebreeding_weight_gain),'_Zkr',num2str(wm)];
et_AB='__';
for iAB=1:size(AlfBet,2)
    et_AB=[et_AB,num2str(AlfBet(1,iAB)),'_']; %#ok
end
etyk_save=[etyk_save,et_AB];
etyk_save=strrep(etyk_save,'.','p');
etyk_save=strrep(etyk_save,'-','m');
if PureCap==1
    AddEtSave='Cap__';
elseif PureCap==0
    AddEtSave='Inc__';
end
etyk_save=[AddEtSave,etyk_save];
PodstList=↙
['''etyk_save'',''zapis_Mean_arriv1'',''zapis_SD1'',''Day_start'',''AlfBet'',''zapi↙
s_N''',...
↙
',''Arriv_popul'',''Hatch_popul'',''Clutchsize_popul'',''Offval_popul'',''zapis_Fe↙
dDistr''',...
↙
',''vect_offval'',''zapis_Day_max'',''LeanBodyMass'',''MaxBodyMass'',''Clutchsize_m↙
ax''',...
↙
',''Reserves_max'',''Condition_maxcat'',''Val_DstGain'',''Prob_DstGain'',''PureCap↙
',''wm'',''wx''',...
↙
',''Day_end'',''Day_max'',''Eggproductioncost'',''Eggweight'',''Incubation'',''Mean↙
```

```
_arriv1'',''Prebreeding_weight_gain'',''SD1'']];

v_trjEgiCnd_lab=[];
for gi1=1:size(AlfBet,2)
    v_trjEgiCnd_lab=[v_trjEgiCnd_lab,'''zapis',num2str(gi1),'_trj_Cond'',''zapis',↙
num2str(gi1),'_trj_egg'',']; %#ok
end
v_trjEgiCnd_lab(end)=[];
PodstList=[PodstList,',',v_trjEgiCnd_lab];
eval(['save(etyk_save,',PodstList,');'])

toc
clearvars -EXCEPT etyk_save

%\\\\\\\\\\\\\\\\\\\\\ PLOTTING \\\\\\\\\\\\\\\\\\\\\
load(etyk_save)
scrsz = get(0,'ScreenSize'); fax=8; flb=11;
hand=figure('Position',[100 100 scrsz(3)/2  scrsz(4)/1.3]);
hg_MPL=0.35;
%Income and Capital Breeding - panels
Pos_ABC=[0.32 0.55 0.4 hg_MPL; 0.32 0.1 0.4 hg_MPL];%main
Pos_clu_ABC= [0.8 0.86 0.18 0.1; 0.8 0.37 0.18 0.1];%clutch size
Pos_ofv_ABC= [0.8 0.71 0.18 0.1; 0.8 0.22 0.18 0.1];%recruitment probability
Pos_recr_ABC=[0.8 0.56 0.18 0.1; 0.8 0.07 0.18 0.1];%recruitment
Pos_food=[0.08 0.63 0.13 0.22; 0.08 0.18 0.13 0.22];%food gain

Pos_ABC_arri=[0.38 0.55 0.25 hg_MPL/2; 0.38 0.101 0.25 hg_MPL/2];
if PureCap==1
    Pos_ABC_bree=[0.57 0.55 0.1 hg_MPL/2;0.57 0.101 0.1 hg_MPL/2];
elseif  PureCap==0
    Pos_ABC_bree=[0.54 0.55 0.1 hg_MPL/2;0.54 0.101 0.1 hg_MPL/2];
end

for glp=1:size(AlfBet,2)
    axes('Position',Pos_food(glp,:));
    Prob_DstGain=zapis_FeedDistr(glp,:,1);
    Val_DstGain=zapis_FeedDistr(glp,:,2);
    bar(Val_DstGain,Prob_DstGain,0.7,'facecolor',[0.4 0.4 0.4],'Edgecolor','none')
    hold on
    set(gca,'Color','none','box','off','fontsize',fax-1)
    set(gca,'xtick',-1:0.5:3,'xticklabel',{-Prebreeding_weight_gain,'',0,'',↙
Prebreeding_weight_gain,'',2*Prebreeding_weight_gain,'',3*Prebreeding_weight_gain})
    xlim([wm wx])
    xlabel({'Food gain \itw_i\rm [g]'},'fontsize',flb-2)
    y_lbs=ylabel('Probability','fontsize',flb-2);
    set(y_lbs,'position', get(y_lbs,'position')-[0.1,0,0])

    axGL=axes('Position',Pos_ABC(glp,:));
    eval(['tr_Cnd=zapis',num2str(glp),'_trj_Cond;'])
    eval(['tr_Egg=zapis',num2str(glp),'_trj_egg;'])
    vct_arr=Arriv_popul(glp,:);
    hold on
    [distr_arr,~]=histcounts(Arriv_popul(glp,:),0:1↙
```

```
zapis_Day_max,'normalization','probability');
    mx_ax_arr=max(distr_arr);
    distr_arr_skal=LeanBodyMass+((distr_arr./mx_ax_arr).*(MaxBodyMass↙
LeanBodyMass))*0.5;
    area(1:1:zapis_Day_max,distr_arr_skal,'Linestyle','none','Facecolor',[0.16 0.35↙
1]);

    [distr_breed,~]=histcounts(Hatch_popul(glp,:),0:1↙
zapis_Day_max,'normalization','probability');
    mx_ax_bree=max(distr_breed);
    distr_breed_skal=LeanBodyMass+((distr_breed./mx_ax_bree).*(MaxBodyMass↙
LeanBodyMass))*0.5;
    if PureCap==1
        area(0:zapis_Day_max-1,distr_breed_skal,'Linestyle','none','Facecolor',[204↙
0 0]./255)
    elseif PureCap==0
        area(0:zapis_Day_max-1,distr_breed_skal,'Linestyle','none','Facecolor',[0↙
0.70 0])
    end
    ax_arri=axes('Position',Pos_ABC_arri(glp,:));
    set(ax_arri,'Color','none','Ycolor',[.16 .35 1],'xtick',[],'fontsize',fax-1)
    ylabel('Frequency','fontsize',fax)
    ylim([0 mx_ax_arr]);

    ax_bree=axes('Position',Pos_ABC_bree(glp,:));
    if PureCap==1
        set(ax_bree,'Color','none','Ycolor',[204 0 0]./255,'xtick',[],'TickLength',↙
[0.025 0],'fontsize',fax-1,'Yaxislocation','right')
    elseif PureCap==0
        set(ax_bree,'Color','none','Ycolor',[0 0.70 0],'xtick',[],'TickLength',↙
[0.025 0],'fontsize',fax-1,'Yaxislocation','right')
    end
    ylabel('Frequency','fontsize',fax)

    ylim([0 mx_ax_bree]);
    axes(axGL) %#ok
    if PureCap==1
        range_of_arriv=round(quantile(Arriv_popul(glp,:),0.5)-1);% arrival dates↙
for plotting
    else
        range_of_arriv=round(quantile(Arriv_popul(glp,:),0.5)+1);
    end

    for q=1:length(range_of_arriv)
        [~,idx_individual]=find(range_of_arriv(q)==vct_arr,1,'first');
        trj_urwana=tr_Cnd(idx_individual,:);
        trj_JajGnzd=tr_Egg(idx_individual,:);
        DayOfFrstEgg=find(trj_JajGnzd>0,1,'first');
        trj_urwana(DayOfFrstEgg:end)=NaN;
        trj_urwana2=NaN(size(trj_urwana));
        trj_urwana2(DayOfFrstEgg-1)=max(trj_urwana);
        if PureCap==1
            plot(trj_urwana2,'ko','Linewidth',0.5,'markerfacecolor',[204 0 0].↙
```

```
/255,'markersize',4)
            plot(trj_urwana,'-','Linewidth',0.75,'Color',[204 0 0]./255)
        elseif PureCap==0
            plot(trj_urwana2,'ko','Linewidth',0.5,'markerfacecolor',[0 0.60↙
0],'markersize',4)
            plot(trj_urwana,'-','Linewidth',0.75,'Color',[0 0.60 0])

        end
    end
    axis([51 209 LeanBodyMass MaxBodyMass+(Reserves_max/Condition_maxcat)*3])
    set(gca,'xtick',0:30:zapis_Day_max,'XTickLabel',↙
{'','Jan','Feb','Mar','Apr','May','Jun','Jul','Aug','Sep','Oct'},...
        'ytick',1200:200:2400,'yticklabel',↙
{'1200','','1600','','2000','','2400'},'fontsize',fax)
    y_lbs=ylabel('Condition [g]','fontsize',flb);
    set(y_lbs,'position', get(y_lbs,'position')-[5,0,0])

    if glp==1
        if PureCap==1
            txAnnot='    \rmFood gain \itw\rm (\color[rgb]{0.4    0.4    0.4}↙
grey\rm\color{black})      Arriving (\color[rgb]{0.16 0.35 1}blue\rm\color{black}↙
Nesting onset, distr. (Capital) (\color[rgb]{0.8 0 0}red\rm\color{black})';
        elseif PureCap==0
            txAnnot='    \rmFood gain \itw\rm (\color[rgb]{0.4    0.4    0.4}↙
grey\rm\color{black})      Arriving (\color[rgb]{0.16 0.35 1}blue\rm\color{black}↙
Nesting onset, distr. (Income) (\color[rgb]{0 0.7 0}green\rm\color{black})';
        end
        annotation('textbox',[.01 .88 .9 .1],'String',↙
txAnnot,'Color','k','Fontsize',flb-1,'Linestyle','none')
    end
    c_lbs=xlabel('Time');
    set(c_lbs,'position', get(c_lbs,'position')-[0,80,0],'fontsize',flb)
    axes(ax_arri) %#ok
    axes(ax_bree) %#ok

    %HISTOGRAM RECRUITMENT PROBABILITY
    axes('Position',Pos_ofv_ABC(glp,:));
    OFFVAL=NaN(1,sum(Clutchsize_popul(glp,:)));
    is=1;
    for it1=1:length(Clutchsize_popul(glp,:))
        CluSiz=Clutchsize_popul(glp,it1);
        OFFVAL(1,is:is+CluSiz-1)=Offval_popul(glp,it1);
        is=is+CluSiz;
    end
    [distr_pop_offval,~]=histcounts(OFFVAL,0:0.05:1.05);
    if PureCap==1
        bar(0:0.05:1,distr_pop_offval/sum(Clutchsize_popul(glp,:)),'barwidth',.↙
4,'facecolor',[204 0 0]./255,'Edgecolor','none');
    elseif PureCap==0
        bar(0:0.05:1,distr_pop_offval/sum(Clutchsize_popul(glp,:)),'barwidth',.↙
4,'facecolor',[0 0.70 0],'Edgecolor','none');
    end
    axis([0,1,0,1])
```

```
    set(gca,'box','off','fontsize',fax-1)
    xlabel('Recruitment probability','fontsize',flb-2)
    ylabel('Frequency','fontsize',flb-2)
    %HISTOGRAM CLUTCH SIZE
    axes('Position',Pos_clu_ABC(glp,:));
    [distr_pop_clutchsize,~]=histcounts(Clutchsize_popul(glp,:),1-0.5:
Clutchsize_max+0.5);
    if PureCap==1
        bar(1:Clutchsize_max,distr_pop_clutchsize./sum
(distr_pop_clutchsize),'facecolor',[204 0 0]./255,'Edgecolor','none');
    elseif PureCap==0
        bar(1:Clutchsize_max,distr_pop_clutchsize./sum
(distr_pop_clutchsize),'facecolor',[0 0.70 0],'Edgecolor','none');
    end
    axis([1-0.5,Clutchsize_max+0.5,0,1])
    set(gca,'box','off','fontsize',fax-1)
    xlabel('Clutch size','fontsize',flb-2)
    %HISTOGRAM RECRUITMENT
    prc=0.25;
    axes('Position',Pos_recr_ABC(glp,:));
    TmRecr=Clutchsize_popul(glp,:).*Offval_popul(glp,:);
    [distr_pop_recr,~]=histcounts(TmRecr,(min(TmRecr)-prc):prc:(max(TmRecr)+prc));
    if PureCap==1
        bar((min(TmRecr)-prc):prc:max(TmRecr),distr_pop_recr./sum
(distr_pop_recr),'facecolor',[204 0 0]./255,'Edgecolor','none');
    elseif PureCap==0
        bar((min(TmRecr)-prc):prc:max(TmRecr),distr_pop_recr./sum
(distr_pop_recr),'facecolor',[0 0.70 0],'Edgecolor','none');
    end
    axis([(min(TmRecr)-prc),(max(TmRecr)+prc),0,max(distr_pop_recr./sum
(distr_pop_recr))*1.2])
    set(gca,'box','off','fontsize',fax-1)
    xlabel('Recruits per female','fontsize',flb-2)
end
set(hand,'PaperUnits','centimeters','Papersize',[16 13],'Paperposition',[0 0 16
13]);
```
